# Suture of Arteries

**Published:** 1909-12

**Authors:** 


**Suture of Arteries.** By E. ARCHIBALD SMITH, M.B., Ch.B. Pp. viii, 70. London: Oxford Medical Publications. 1909.

Many think that the suture of arteries is quite a new surgical method. It is rather startling to find from the copy of a letter which the author inserts that it was used in 1759, and by the use of a pin rather more than a quarter of an inch in length, around which the suture was twisted. Indeed, the opening in the artery was closed as a hare-lip wound was united some years ago. It healed well and the circulation continued through the artery. The wound in the artery was made to cure a small traumatic aneurism, and the case is referred to in a letter to

John Hunter. Mr. Archibald Smith gives us an historical summary of the operation of suture of arteries, and describes various methods which different surgeons have adopted. He also gives us the account of some operations he has himself performed on animals in Vienna, and the description of some of the histological appearances shown in sections of the healed and healing arteries. He prefers to have the artery slightly on the stretch laterally, during the suturing, which he does either with quilled sutures over catgut or with a series of a kind of single mattress stitch. He uses a pair of small pressure forceps to control the circulation, but he does not grasp the artery with them, he only uses them as a support for a rubber band. Two questions naturally arise in connection with this method of suturing arteries: (1) What is the risk of hemorrhage, and (2) What is the risk of occlusion of the vessel lumen? The first risk seems really very small. The sheath of the vessel and the supporting fascia should be sutured as well as the vessel wall. Archibald Smith's sutures were fine silk on round needles, and penetrated all the coats. He thinks thrombosis always occurs on the portion of suture which penetrates the intima, but does not spread so as to occlude the vessel lumen.

We think this little book is a very useful one, and will well repay careful perusal.

